# The role of fatigue in patients with complex regional pain syndrome

**DOI:** 10.1007/s00415-024-12473-3

**Published:** 2024-06-08

**Authors:** Matthias Wiemann, Sarah-Luis Blendow, Nikolas Zimowski, Elena Enax-Krumova, Robert Fleischmann, Iris-Katharina Penner, Matthias Grothe, Sebastian Strauss

**Affiliations:** 1 Department of Neurology, University Medicine Greifswald, Ferdinand-Sauerbruch-Str. 1, 17475 Greifswald, Germany; 2 Department of Neurology, BG University Hospital Bergmannsheil gGmbH, Ruhr University Bochum, Bochum, Germany; 3 Department of Neurology, Inselspital, Bern University Hospital, University of Bern, Bern, Switzerland

**Keywords:** CRPS, Complex regional pain syndrome, Fatigue

## Abstract

**Background and Purpose:**

Fatigue affects patients across a variety of neurological diseases, including chronic pain syndromes such as complex regional pain syndrome (CRPS). In CRPS, fatigue is often underestimated, as the focus lies in the assessment and managing of pain and sensorimotor deficits. This study aimed to investigate the prevalence, characteristics, and influence of fatigue on CRPS severity and quality of life in these patients. Such insights could enhance the clinical management of this challenging condition.

**Methods:**

In this prospective study, 181 CRPS patients and 141 age and gender-matched individuals with injury but without chronic pain were interviewed using the Fatigue Scale for Motor and Cognitive Function to assess fatigue. Depressive symptoms and quality of life (QoL) were also evaluated as additional outcome measures. Statistical analysis was performed to examine differences in fatigue prevalence between the groups, as well as associations with CRPS severity, pain levels, and clinical phenotype. In addition, best subsets regression was used to identify the primary factors influencing QoL. Fatigue was tested in a mediation analysis as a mediator between pain and depression.

**Results:**

CRPS patients showed significantly higher fatigue levels compared to controls (CRPS: 75 [IQR: 57–85] vs. controls: 39 [IQR: 25–57]). Based on the FSMC, 44.2% in the control group experienced fatigue, while 85% of patients with CRPS experienced fatigue (*p* < 0.001), of which 6% were mild, 15% moderate, and 67% severe. In CRPS severe fatigue was associated with higher pain intensities compared to no fatigue (pain at rest: *p* = 0.003; pain during movement: *p* = 0.007) or moderate fatigue (pain during movement: *p* = 0.03). QoL in our cohort was mainly influenced by pain (pain during movement: adj.R^2^ = 0.38; *p* < 0.001, pain at rest: Δadj.R^2^ = 0.02, *p* = 0.007) and depressive symptoms (Δadj.R^2^ = 0.12, *p* < 0.001). Subsequent analyses indicated that pain and depressive symptoms primarily impact QoL in CPRS whereas fatigue may exert an indirect influence by mediating the connection between pain and depression (*p* < 0.001).

**Conclusions:**

This pioneering study investigates the prevalence of fatigue in CRPS patients and its relation to disease characteristics. Our results indicate a high prevalence of severe fatigue, strongly correlated with pain intensity, and its importance in the interaction between pain and depression in CRPS. These findings underscore the significant role of fatigue as a disease factor in CRPS. Therefore, the evaluation of CRPS-related disability should include a standardized assessment of fatigue for comprehensive clinical management.

## Introduction

Chronic fatigue is a typical symptom of several neurological diseases, e. g. multiple sclerosis, postpoliomyelitis, chronic inflammatory demyelinating polyneuropathy and after stroke [1–3]. However, fatigue seems to be highly prevalent also in chronic pain conditions like headache disorders, fibromyalgia and chronic low back pain [4, 5]. Fatigue has been reported to follow pain onset [6–8] and to decrease after improvement in pain [9]. Finally, pain experience and the level of fatigue seem to be associated indicating a possible etiological relationship [5]. The ongoing inflammation and autonomic dysfunction as well as mechanisms of central sensitization have been discussed as relevant pathophysiological aspects in the development of fatigue [10–12].

Fatigue acts as a mediator in the relationship between pain and depression [13] and has a considerable direct and indirect impact on the reduced quality of life in patients [14]. Consequently, addressing fatigue is often difficult in clinical practice [15] and has notable implications for healthcare costs [16].

Complex regional pain syndrome (CRPS) is a challenging chronic pain condition that is often caused by an injury of the limb, including strong pain disproportionate to the inciting event, alterations of sensory perceptions and disturbed motor function [17]. In addition to regional symptoms, 65% of CRPS patients present with neuropsychological symptoms, such as deficits in memory and concentration or “neglect-like” symptoms, despite the absence of any brain lesion [18–20]. Further, patients with chronic CRPS frequently report general symptoms such as lethargy, tiredness, and weakness, all of which are facets of fatigue [21]. Surprisingly, data on the prevalence of motor and cognitive fatigue and their associations to clinical features of CRPS are scarce but of high clinical relevance, as fatigue often interferes with patients’ activities of daily living and has a remarkable negative impact on quality of life in pain patients in general [22].

Thus, we conducted a cross-sectional survey using a multidimensional assessment approach within a national German cohort comprising 200 individuals diagnosed with Complex Regional Pain Syndrome (CRPS) alongside a control group of patients with a history of limb injury but without chronic pain. This assessment included the screening for fatigue utilizing an established rating instrument, evaluation of CRPS phenotype and severity, and application of other patient-reported outcomes including quality of life, depressive and anxiety symptoms.

We hypothesized that patients with CRPS have a higher prevalence and severity of overall fatigue compared to those without chronic pain (H1). Furthermore, we expected a high rate of severe fatigue in CRPS patients (H2), and a direct correlation between fatigue severity, pain intensity, and the severity of CRPS symptoms (H3). We assumed a stronger correlation between motor fatigue and levels of movement-related pain due to greater involvement of the motor system (H3.1). We furthermore expected more pronounced fatigue in patients demonstrating a predominant central CRPS phenotype [12] (H4). Additionally, we examined the association between fatigue and quality of life (H5) [14] as well as depressive symptoms (H6) [13].

## Methods

### Study design and patient recruitment

Patients suffering from CRPS were prospectively recruited for a cross-sectional survey through specialized pain centers and by making contact via patient support groups between 03/2020–09/2021. The diagnosis had to be either confirmed by the treating pain center, or patients recruited through support groups had to specify the place and date of diagnosis, as well as the triggering event. Further, the International Association for the Study of Pain (IASP) diagnostic criteria for CRPS were also assessed in the medical history and reviewed before study inclusion [23]. Further inclusion criteria was age between 18 and 70 years. Given the exploratory nature of the study, all individuals meeting these criteria were initially included in the analysis. However, participants with incomplete information regarding CRPS and fatigue severity were subsequently excluded from the final analysis. This approach ensured that only datasets containing comprehensive information were considered for the study’s objectives.

As a control group, we recruited subjects from the local trauma centers between 01/2023 and 03/2023 who suffered an injury to the upper or lower extremities in the past but did not subsequently develop CRPS. Inclusion criteria were fracture or invasive intervention of the lower or upper limbs and age between 18 and 70 years.

**Fig. 1:**
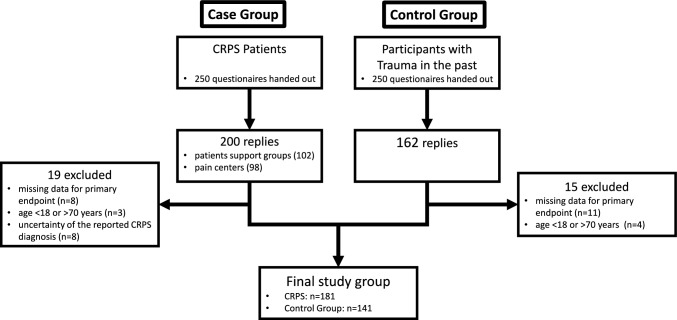
Flowchart of study inclusion and final group size

After handing out 250 questionnaires each, we received 200 questionnaires from CRPS patients (*n* = 102 from support groups, *n* = 98 from specialized pain centers) and 162 from patients with injury of a limb who did not develop CRPS, which were screened for completeness of the data and inclusion criteria. (Fig. 1). (for more details on patient’s characteristics, as well as the structure and content of the questionnaire see) [24].

The study was approved by the ethics committee of the University of Greifswald, Germany (BB176/20) and the local ethics committee of the participating specialized pain center in Bochum (reg. nr. 21-7165). The study was prospectively registered in the German Clinical Trials Register (DRKS00022961).

### Assessment of demographic characteristics and patient-reported outcome measurements

All included patients received a standardized questionnaire to assess their sociodemographic characteristics.

To record relevant comorbidities such as depression, anxiety, and quality of life, the Hospital Anxiety and Depression Scale (HADS) [25] and the EuroQol five-dimension five-level (EQ-5D-5L) [26, 27] were part of the questionnaire.

The HADS [25] was used to self-report and rate the severity of depressive (HADS-D subscale) and anxiety (HADS-A subscale) symptoms during the past week. Each subscale consists of seven questions with a four-point Likert scale, total scores per subscale range from 0 to 21 with higher scores indicating more severe depression and anxiety.

The EQ-5D-5L measures five dimensions mobility, self-care, usual activities, pain/discomfort and anxiety/depression at levels of having no, slight, moderate, severe problems, or being unable to do a task and ranges from 1 (full health) to -0.27 (worse than death) [26].

### Assessment of the primary endpoint—Fatigue

All participants were investigated using the Fatigue Scale for Motor and Cognitive Function (FSMC): The 20-item comprehensive, self-report questionnaire was developed by Penner et al. in 2009 [28] and assesses cognitive and physical fatigue with two subscales (range per subscale: 10–50). Patients can select one of five options (“Strongly disagree” to “Strongly agree”). For the subscale of cognitive and physical fatigue, scores above 22 were defined as “mild”, above 28 as “moderate”, and above 34 as “severe” fatigue.

### CRPS-specific assessments

Typical CRPS symptoms and medical history, disease onset, as well as the place of diagnosis were surveyed. Clinical characteristics of CRPS were captured according to the new IASP diagnostic criteria and were used to enhance the reliability of diagnosing CRPS [23]. A visual analogue scale (VAS, 10 cm) assessed current pain during movement (movement pain) and pain at rest. The severity of the disease (including questions on sensory, vaso/sudomotor, motor and trophic dysfunction) was evaluated using an adaptation (only self-reported symptoms) of the validated CRPS severity score (CSS) [29]. Classification of CRPS patients in clinical phenotypes (“peripheral”, “mixed”, and “central”) was carried out based on the reported diagnostic criteria referring to a recently proposed algorithm [30]. In this algorithm minor injury eliciting CRPS, motor signs, allodynia, and glove/stocking-like sensory deficits reflecting the central phenotype whereas symptoms like edema, skin color changes, skin temperature changes, sweating, and trophic changes, predominantly represent the peripheral phenotype [30].

### Statistics

The descriptive data reported here are part of a more extensive investigation of comorbidities in patients with CRPS. Therefore, there was no à priori sample size calculation for the possible association of fatigue and CRPS (for more details see [31]).

All analyses were done using R, version 4.2.2. Correlations were calculated using the Pearson product-moment correlation coefficient. To test differences of means between groups, one-way analysis of variance (ANOVA) and Tukey’s post-hoc test. Independent variable in the ANOVA model was fatigue severity. When prerequisites for an ANOVA were not met (i.e. heteroscedasticity, non-normal distribution), we used a Kruskal–Wallis rank sum test with a post-hoc Dunn’s test and Holm correction instead. When comparing the means of the two groups, we used Student’s *t* test or Wilcoxon rank sum test depending on the distribution. For categorical data and frequencies, we used the Chi-squared test or Fisher’s exact test if fewer than 5 observations per cell existed. If indicated, group mean with 95%-confidence interval (CI) is given for the parametric test and a median with interquartile range (IQR) for non-parametric tests. As a robust method for linear regression, we utilized MM-type estimators with bi-square redescending score function [32, 33].

Multiple regression was used to measure the various factors influencing QoL. To avoid overfitting, variables were first selected using a best-subset regression. In the best subset regression, all variables are tested in all combinations as predictive of the dependent variable. Adjusted R^2^ (adj. R^2^), Bayesian information criterion (BIC), and Mallow’s Cp are calculated for each combination. Models were sorted by highest adj. R^2^, lowest BIC, and the lowest difference between Mallow’s Cp and a number of the regressor. Model selection was based on BIC and Mallow’s Cp with adj. R^2^ as a subsequent criterion. Dependent variables included for best subset regression were CRPS duration, sex, movement pain, pain at rest, CSS, HADS depression score, HADS anxiety score, and severity of fatigue. To evaluate the effect of fatigue on the development of depression in the context to chronic pain mediation analyses were performed using the mediation package [34]. Here ordinary least squares regression, with z-standardized (β) and unstandardized path coefficients (B) for all effects (total, direct, and indirect) were used. Age, sex, and month since CRPS onset were added as control variables. Non-parametric bias-corrected and accelerated bootstrapping with 10,000 samples was employed to compute the confidence intervals [35, 36].

## Results

The total study population included in the statistical analysis consisted of 328 participants (228 female, mean age 47.1 ± 12.0 years). There was no significant difference in age (*p* = 0.60) or sex (*p* = 0.38) between the CRPS (*n* = 181) and control group (*n* = 147). There were no significant differences in CRPS characteristics between patients recruited from support groups and patients recruited from specialized pain centers. Twenty-five patients (14%) showed predominantly peripheral symptoms and could therefore be classified as peripheral phenotypes, whereas 67 patients (37%) had mainly symptoms presumably related to central reorganization. 89 patients (49%) could be categorized as “mixed” phenotype.

### Fatigue comparison between trauma patients with and without CRPS and fatigue characteristics in CPRS patients (H1 & H2)

**Fig. 2:**
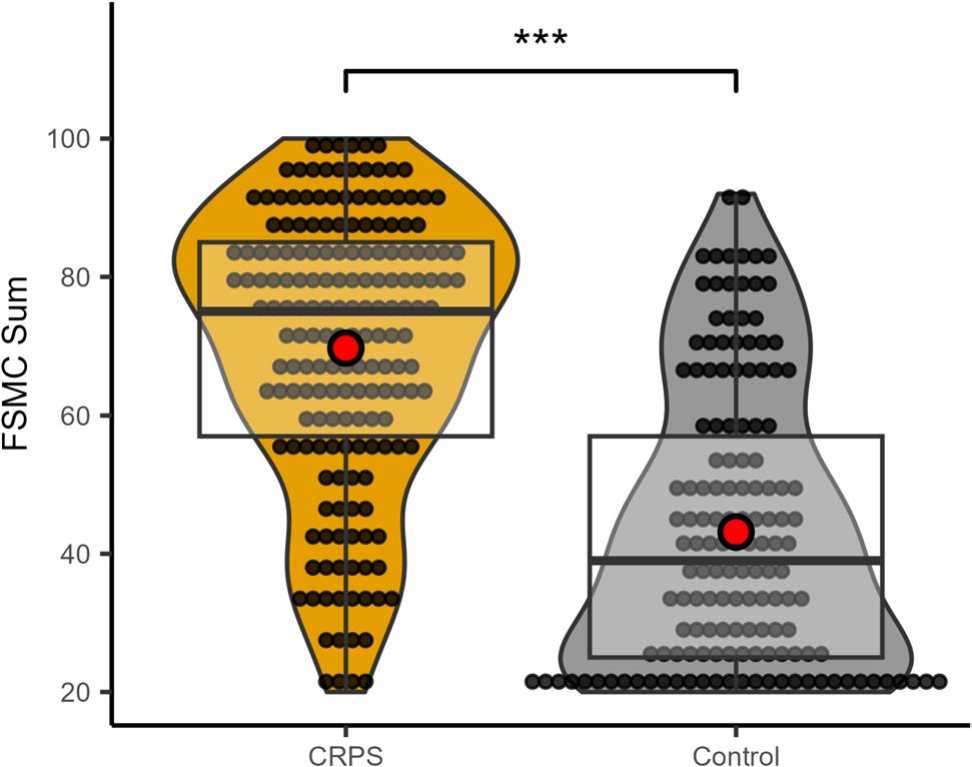
Group difference between CRPS patients and patients with injury of a limb only. Illustrated as box plots with the upper and lower quartile (black square), group median (bold black line), and mean (red dot). ***indicates a significance level of *p* < 0.001

CRPS patients reported a median of 75 [IQR: 57–85] points on the FSMC scale, while the control group reported a median of 39 [IQR: 25–57] points. This difference was significant (W_181,141_ = 21,676, *p*-value < 0.001). Based on the FMSC, 85.1% of patients with CRPS and 44.2% of people with injury only reported fatigue (chi-squared (1) = 59.23, *p* < 0.001). *Among CRPS patients, the most common fatigue level was severe (67.4%), followed by no fatigue (14.9%), moderate fatigue (12.7%), and mild fatigue (5.0%). The HADS depression scores (F(3, 177)* = *24.20, p* < *0.001) and anxiety scores (F(3, 177)* = *23.16, p* < *0.001), as well as pain scores, varied with the severity of fatigue, with higher scores corresponding to more severe fatigue. There were also group differences for QoL (F(3, 167)* = *11.09, p* < *0.001) and the Healthscale (F(3, 175)* = *15.51, p* < *0.001), with CRPS patients experiencing higher fatigue reporting lower scores. See *Table 1* for further details on the differences between fatigue levels.* An MM-type estimator linear regression model with all gathered treatments (see Table 1 below for a list) showed only a significantly higher FSMC sum score if a spinal cord stimulator was present (11.46 ± 4.23, t (174) = 2.68, *p* = 0.008) (Fig. 2).

**Table 1:** Overview of patients’ characteristics sorted according to fatigue severity measured with the FSMC, *p* indicates differences between fatigue groups using ANOVA for continuous data and Fisher’s exact test for categorical data

	Overall	No Fatigue	Mild fatigue	Moderate fatigue	Severe fatigue	*p*
*n* (%)	181 (100)	27 (14.9)	9 (5.0)	23 (12.7)	122 (67.4)	
Age (mean (SD))	46.80 (12.06)	44.81 (12.98)	48.33 (17.04)	44.00 (13.26)	47.66 (11.21)	0.438
Sex = female (%)	129 (72.9)	16 (61.5)	7 (77.8)	16 (76.2)	90 (74.4)	0.561
Month since CRPS onset (median (IQR))	36.00 [12.00, 90.00]	24.00 [12.00, 36.00]	48.00 [12.00, 72.00]	36.00 [12.00, 66.00]	36.00 [24.00, 96.00]	0.016†
*Limb* *n*, (%)						0.397
Upper	110 (60.8)	20 (74.1)	8 (88.9)	13 (56.5)	69 (56.6)	
Lower	63 (34.8)	6 (22.2)	1 (11.1)	9 (39.1)	47 (38.5)	
Both	8 ( 4.4)	1 ( 3.7)	0 ( 0.0)	1 ( 4.3)	6 ( 4.9)	
*Phenotype* *n*, (%)						0.505
Peripheral	25 (13.8)	4 (14.8)	1 (11.1)	5 (21.7)	15 (12.3)	
Mixed	89 (49.2)	14 (51.9)	2 (22.2)	11 (47.8)	62 (50.8)	
Central	67 (37.0)	9 (33.3)	6 (66.7)	7 (30.4)	45 (36.9)	
Pain at rest (mean (± SD))	4.82 (2.39)	3.56 (3.03)	4.11 (1.67)	4.46 (2.19)	4.82 (2.39)	0.007
Movement pain (median (IQR))	7.00 [5.88, 8.00]	6.00 [4.25, 7.00]	6.50 [5.50, 7.50]	6.00 [4.00, 7.50]	7.50 [6.00, 8.50]	0.002†
HADS depression score (mean (± SD))	8.90 (4.87)	3.70 (2.63)	4.44 (3.13)	8.13 (3.88)	10.52 (4.49)	< 0.001
HADS anxiety score (mean (± SD))	8.69 (4.51)	4.37 (3.16)	4.67 (2.45)	6.87 (3.97)	10.28 (4.04)	< 0.001
QoL (mean (± SD))	0.45 (0.31)	0.67 (0.30)	0.69 (0.29)	0.54 (0.26)	0.36 (0.28)	< 0.001
Healthscale (mean (SD))	47.15 (20.56)	65.65 (18.89)	58.78 (17.17)	54.04 (17.40)	41.00 (18.59)	< 0.001
CSS (median [IQR])	7.00 [6.00, 8.00]	6.00 [4.50, 7.00]	7.00 [6.00, 7.00]	7.00 [6.00, 8.00]	7.00 [6.00, 8.00]	0.263†
Any treatment *n*, (%)	156 (86.2)	22 (81.5)	9 (100.0)	21 (91.3)	104 (85.2)	0.589
Any medication *n*, (%)	138 (76.2)	18 (66.7)	9 (100.0)	14 (60.9)	97 (79.5)	0.046
NSAID *n*, (%)	74 (40.9)	11 (40.7)	4 ( 44.4)	7 (30.4)	52 (42.6)	0.757
Opiod *n*, (%)	106 (58.6)	11 (40.7)	7 ( 77.8)	11 (47.8)	77 (63.1)	0.076
AED *n*, (%)	112 (61.9)	13 (48.1)	8 ( 88.9)	12 (52.2)	79 (64.8)	0.103
Any non-medication intervention *n*, (%)	130 (71.8)	19 (70.4)	7 ( 77.8)	17 (73.9)	87 (71.3)	1.000
PT / OT *n*, (%)	117 (64.6)	18 (66.7)	7 ( 77.8)	15 (65.2)	77 (63.1)	0.882
SCS *n*, (%)	22 (12.2)	2 ( 7.4)	0 ( 0.0)	1 ( 4.3)	19 (15.6)	0.320
Acupuncture *n*, (%)	19 (10.5)	2 ( 7.4)	1 ( 11.1)	3 (13.0)	13 (10.7)	0.916

^†^Kruskal–Wallis test was performed n number, *SD* standard deviation, *IQR* interquartile range, *HADS* Hospital anxiety and depression scale, *QoL* Quality of life, *CSS* CRPS severity score, *NSAID* Nonsteroidal anti-inflammatory drug, *AED* Antiepileptic drug, *PT* Physical therapy, *OT* occupational therapy, *SCS* Spinal cord stimulator

### Relationship of fatigue and CRPS severity and pain levels (H3)

### CRPS severity score

**Fig. 3:**
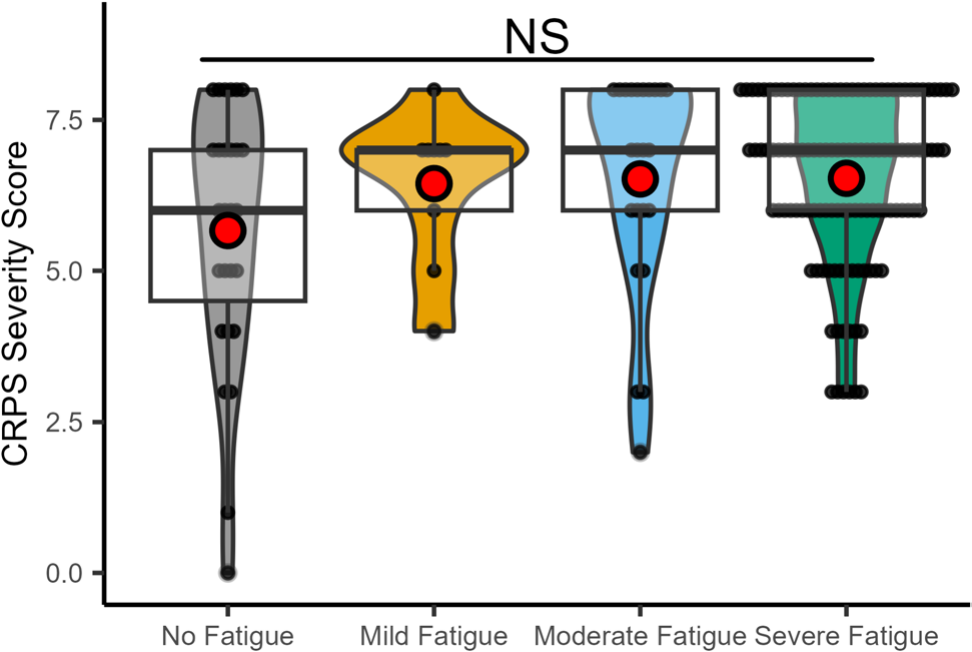
Relationship of fatigue severity and CRPS severity score illustrated as box plots with the upper and lower quartile (black square), group median (bold black line), and mean (red dot). *NS* not significant

Kruskal–Wallis test showed no significant difference between groups (chi-squared (3) = 3.98, *p* = 0.26; Fig. 3).

### Pain intensities

**Fig. 4:**
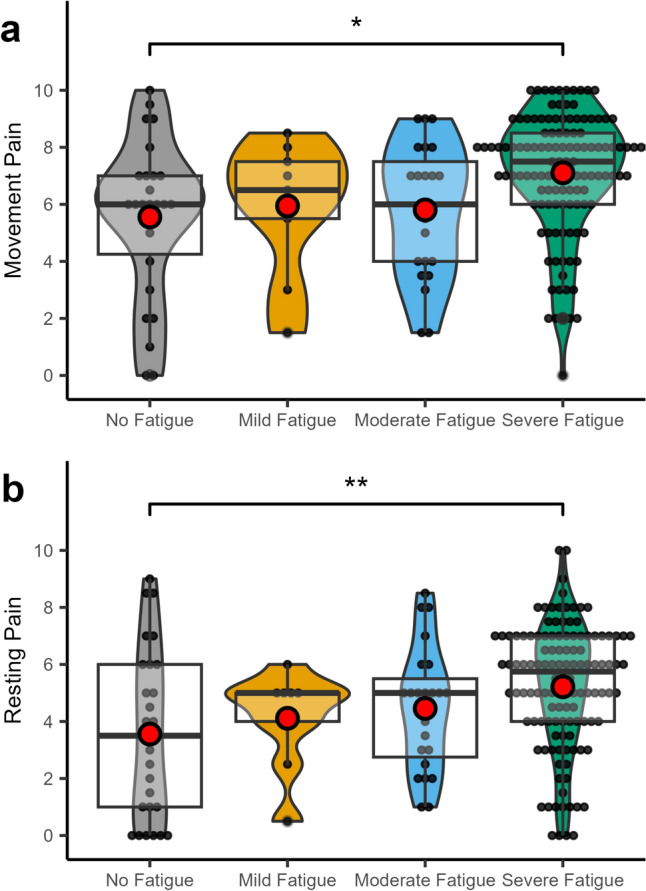
Differences of movement (**a**) and pain at rest (**b**) at different fatigue levels in CRPS patients illustrated as box plots with the upper and lower quartile (black square), group median (bold black line), and mean (red dot). *indicates a significance level of *p* < 0.05; **indicates a significance level *p* < 0.01

Rating of movement pain and pain at rest differed between the groups (movement pain: chi-squared (3) = 14.6, *p* = 0.002; pain at rest F (3,176) = 4.21, *p* = 0.007). Post hoc statistics revealed that patients with severe fatigue reported significantly higher pain levels at rest (5.22 [CI 4.80–5.63] vs. 3.56 [CI 2.66—4.46], *p* = 0.006) and on movement (7.00 [IQR: 5.88–8.00] vs. 7.50 [IQR 6.00–8.50], *p* = 0.015) compared to patients without. Results are summarized in Fig. 4.

### The motor fatigue subscale best reveals difference in movement pain. (H3.1)

**Fig. 5:**
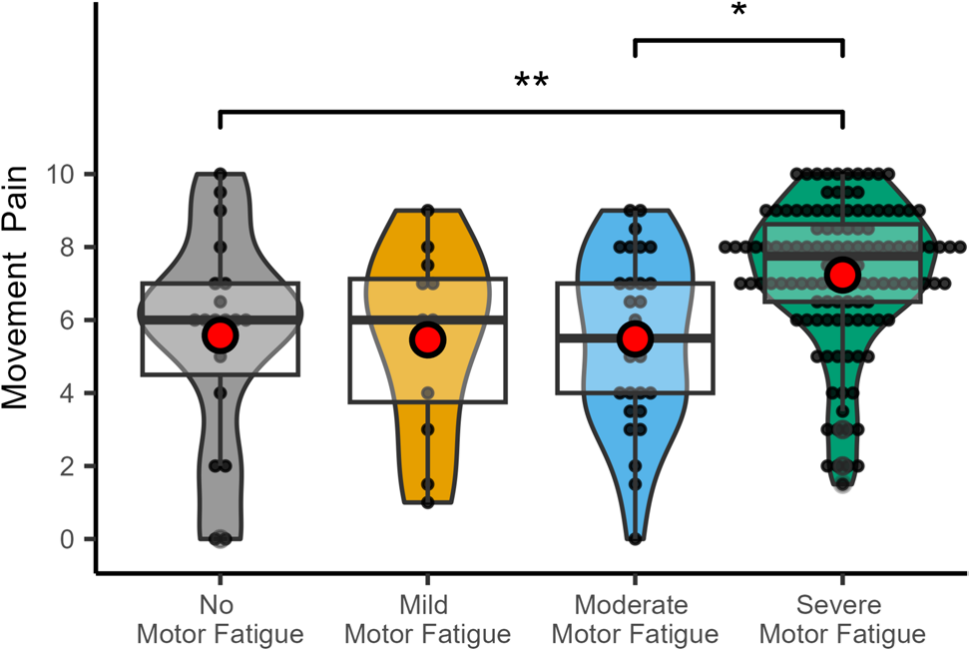
Relationship of motor fatigue severity and movement pain in CRPS patients illustrated as box plots with the upper and lower quartile (black square), group median (bold black line), and mean (red dot). *indicates a significance level *p* < 0.05

When evaluating the association between the severity of motor fatigue and movement pain the Kruskal–Wallis test revealed a significant association (chi-squared (3) = 21.26, *p* < 0.001). Post-hoc analysis revealed significant differences between no motor fatigue (*p* = 0.028) and moderate motor fatigue (*p* = 0.002) compared to the severe fatigue group (Fig. 5).

When calculating the effect of fatigue on movement pain using the FSMC sum (η^2^_H_ = 0.07, *p* = 0.002) as well as the cognitive (η^2^_H_ = 0.06, p = 0.005) and motor domain only (η^2^_H_ = 0.10, *p* < 0.001), the motor domain alone yielded highest Eta[_H_]^2^, indicating the best fit to explain the variance of movement pain.

### Association of fatigue and CRPS phenotype (H4).

Chi-squared test revealed no significant differences in the severity of fatigue and phenotype of CRPS (chi-squared (6) = 5.27, *p* = 0.51).

### Impact of fatigue on patients’ quality of life (H5)

FSMC score and QoL were significantly correlated (*r* =  – 0.47, *p* < 0.001).

In linear regression, a significant effect of fatigue severity on quality of life was only found for severe fatigue (β =  – 0. 30, *p* < 0.001) compared with no fatigue.

The best subsets regression identified the multiple variable model best predicting QoL in CRPS patients. Using the adj. R^2^-value criterion a model consisting of 6 predictors would be preferred (movement pain, HADS depression score, pain at rest, severe fatigue, HADS anxiety score, CRPS duration). When using Mallows’ Cp-statistics, a model with four predictors (movement pain, HADS depression score, pain at rest, severe fatigue) and using BIC, a model with three predictors (movement pain, HADS Depression Score, pain at rest) is preferred.

The best six predictor model and four predictor model regression analysis showed only marginal higher adjusted R^2^-value compared to the three predictor model (0.522 vs. 0.521 vs. 0.519) and did not show a significant difference (F(5) = 1.18, *p* = 0.32 and F(3) = 1.22, *p* = 0.30). It is therefore not justified using the larger six or four-predictor model over the simpler, smaller three-predictor model with movement pain, HADS Depression Score, and pain at rest (Table 2).

**Table 2:** Linear regression with QoL as dependent variable

Influence on quality of life					
	Standardized coefficient	Std. error	Coefficient	Std. error	*p*-value
(Intercept)	0.4533	0.0161	1.101	0.054	< 0.001***
Movement pain	– 0.1130	0.0262	– 0.048	0.011	< 0.001***
HADS depression score	– 0.1042	0.0175	– 0.021	0.004	< 0.001***
Pain at rest	– 0.0719	0.0261	– 0.030	0.011	< 0.01**

Adjusted R^2^ = 0.52, *p* < 0.001

Model parameters of the final model determined by the best subset regression. For standardized coefficients, the predictors have been scaled to account for different range, of scales.

### Impact of pain on depression through fatigue as mediator (H6)

**Fig. 6:**
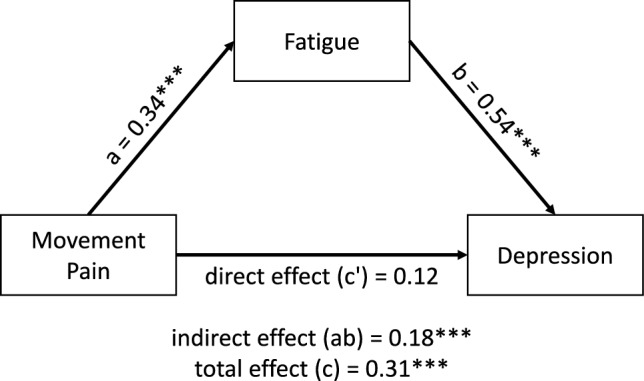
Mediation pathways and coefficients. Difference between the total effect and the sum of direct and indirect effect is due to rounding error. All variables have been z-standardized to account for different ranges of scales; ***indicates a significance level *p* < 0.001

In the mediation analysis, a total effect of pain on depression was observed, (β_c_ = 0.31 [CI 0.16–0.44], B_c_ = 0.64, *p* < 0.001). After entering fatigue as the mediator into the model, movement pain predicted the mediator significantly (β_a_ = 0.34 [CI 0.20–0.47], B_a_ = 2.98, *p* < 0.001), which in turn predicted depression significantly (β_b_ = 0.54 [CI 0.41–0.63], B_b_ = 0.13, *p* < 0.001). The direct effect of pain on depression within the mediation was not significant (direct effect β_c’_ = 0.12 [CI  – 0.01 to 0.25], B_c’_ = 0.26, *p* = 0.074), while the relationship between movement pain and depression was mediated by fatigue significantly (indirect effect β_ab_ = 0.18 [CI 0.10–0.28], B_ab_ = 0.39, *p* < 0.001). For a graphical representation of the results, see Fig. 6. None of the included control variables (age, sex, month since CRPS onset) showed a significant influence in any path.

## Discussion

Our survey included a large national cohort of trauma patients with CRPS and without CRPS. We investigated the prevalence of fatigue, its relationship to CRPS severity as well as the impact on patients’ quality of life for the first time systematically.

Although fatigue also was present in individuals who had experienced injury of a limb but did not develop CRPS, our findings indicated a notably higher prevalence and severity of fatigue among CRPS patients. They exhibited a consistently high prevalence of severe fatigue, which was strongly correlated with the intensity of perceived pain. Furthermore, fatigue significantly mediated the relationship between pain and depression. Remarkably, this fatigue seemed unrelated to the specific CRPS phenotype. Finally, we here propose the FSMC as a valid fatigue screening tool in CRPS that might be easily applicable in clinical routine and scientific context to assess both cognitive and physical aspects of fatigue.

### Implications of the association of fatigue and CRPS for shared mechanisms

CRPS is a disease that is accompanied by persistent, often severe pain in the affected limbs. The high prevalence of fatigue in patients with CRPS confirms previous data of co-occurrence of pain and fatigue in the general population [37] as well as in patients with fibromyalgia [38] and multiple sclerosis [39]. It also indicates potential overlapping pathogenic mechanisms between chronic pain and fatigue [40]. The finding of higher pain levels associated with greater fatigue in our cohort of patients with CRPS has also been described in various diseases accompanied by pain (review [5]:) corroborating the presumption of a direct etiological relationship between pain and fatigue.

In detail, inflammatory processes have been discussed to be the pathophysiological link between fatigue and pain. Complex interactions between increased levels of pro-inflammatory cytokines, alterations in peripheral (peripheral sensitization) and central nervous system (central sensitization) as well as dysregulation of the endocrine system (hypothalamic–pituitary–adrenal axis, resistance to glucocorticoids) have been described in fatigue but also in CRPS [40–45]. Interestingly, the role of dysregulated pro-inflammatory cytokines like interleukin-6 in long-term fatigue is highly topical and of interest since it has been discussed to be a potential link between COVID-19 infection and the resulting high rate of long-term neuropsychiatric symptoms such as fatigue, sleeping difficulties, depression, and anxiety [46].

Central sensitization is discussed as a key mechanism in the pathophysiology of chronic CRPS, especially in the predominant central phenotype (according to the algorithm proposed by Dimova et al. 2020 [30]). There is increasing evidence that central sensitization is also an important factor in the development and maintenance of fatigue independent of pain [12, 47]. Nevertheless, there was no association between fatigue and CRPS phenotype in our questionnaire-based study. Future studies are needed to systematically assess the CRPS phenotype using clinical and neurophysiological methods, particularly in the context of comorbidities, to further evaluate this aspect.

### Interaction of fatigue on quality of life, pain, and depression in CRPS

The Quality of life in our cohort of CRPS patients is mainly driven by pain intensity, in particular movement pain, and depressive symptoms. Fatigue and duration of CRPS as independent factors may only play an additional subordinate role. Pain and affective disorders are of high interest in patients with CRPS since they are highly prevalent and are directly associated with patient’s disease burden [48]. Our results corroborate this clinical relevance and are well in line with existing literature on various pain conditions [49, 50].

Although fatigue exhibits a moderate correlation with Quality of Life (QoL) independently, this correlation diminishes when incorporating the impact of pain and depression on QoL as a subsidiary factor [51]. This contradicts findings in other pain conditions, potentially stemming from the inclusion of depression as an additional predictor. However, the results presented here show for the first time that fatigue acts as a mediator in the relationship between chronic pain and depression in CRPS, and are consistent with existing literature, including studies on multiple sclerosis sclerosis [13]. Therefore, the clinical relevance of fatigue may go underrecognized when assessed along with pain and depressive symptoms.

However, fatigue is often difficult to treat and is barely an objective in CRPS therapy concepts. Specific therapy approaches for fatigue can include cognitive behavioral therapy and graded exercise therapy [52, 53]. At present, there are no pharmaceutical interventions recommended for the treatment of fatigue in CRPS, primarily due to the absence of clinical trial investigations. However, insights from patients with multiple sclerosis indicate that addressing depression through treatment may lead to a decrease in the severity of fatigue [54]. Further investigations on the treatment of fatigue in patients with CRPS are needed to evaluate the effectiveness of different therapy strategies and their impact on the quality of life in these chronic pain condition.

## Limitations

While only half of our patients were recruited from specialized pain centers, there were no discernible differences in relevant clinical data between these patients and those recruited via patient support groups. Nevertheless, uncertainties persist regarding the diagnosis of CRPS, as well as in assessing disease severity and clinical phenotype, particularly given the importance of thorough clinical examination in this context. Moreover, we were not able to capture all medications and their exact dosage that subjects may have been taking. This could be of interest since it is suggested that centrally acting medications generally may have a negative influence on cognitive fatigue levels of individuals with multiple sclerosis [55], whereas antidepressants are discussed to have a positive effect on the individual and thereby have the potential to reduce fatigue [56]. In our cohort. CRPS patients treated with an SCS for pain reported even higher fatigue. It should be kept in mind though that SCS is often used in severe cases of CRPS when other options are not sufficient, and therefore the fact that this is a severely affected patient group could be the reason for this correlation. Future studies should investigate the relationship between fatigue and class of medication, dosage, and duration of intake as well as non-medical treatment more systematically.

Caution is warranted when inferring causal relationships through mediation analysis. The cross-sectional design of this study precludes the establishment of temporal preempts among variables, limiting our ability to discern the directionality of effects. It is important to acknowledge the potential bidirectional nature of the relationship between depression and fatigue, wherein each may exacerbate the other over time. A longitudinal study is needed to better distinguish between cause and effect and the potential magnitude of bidirectionality. Additionally, our mediation analysis solely focused on movement pain, overlooking the potential influence of other variables on the proposed mediation pathways. Consequently, while our findings provide valuable insights, they should be interpreted within the confines of these methodological limitations.

Only a few participants showed mild to moderate fatigue symptoms which statistically underpowers those groups and increases the chance for error. For future sample designs, it should be taken into account that a majority of CRPS patients may already have severe fatigue symptoms.

Finally, caution is required when interpreting the results, since questionnaire studies are subject to sampling and assessment biases, as well as recall and volunteer bias. Further longitudinal studies with thorough clinical examinations, standardized symptom assessment such as e.g., quantitative sensory testing, neurophysiological measurements and neuropsychological assessment as well as assessment of fatigability are needed to clarify both shared pathophysiology aspects and associations of disease progression and development of fatigue [57].

## Conclusions

Severe fatigue is a prevalent symptom in patients with CRPS and is associated with pain intensity. Hence clinical assessment of fatigue in addition to the Budapest criteria should be included as a standard examination for CRPS. We propose the use of the FSMC enabling to describe cognitive and physical aspects of fatigue separately.

## Data Availability

The datasets used and/or analyzed during the current study are available from the corresponding author upon reasonable request.
